# Supplementary material for: Calcareous dinoflagellate blooms during the Late Cretaceous ‘greenhouse’ world—a case study from western Ukraine
Source: PeerJ. 2023 Oct 5;11:e16201. doi: 10.7717/peerj.16201 (PMC10560496; doi:10.7717/peerj.16201)
Supplement: Supplemental Information 1 [file peerj-11-16201-s001.docx]

| Assemblage | | Species/Sample | *Bonetocardiella conoidea* | *Pithonella cardiformis* | *Pihonella lamellata* | *Pithonella ovalis* | *Pithonella sphaerica* | *Stomiosphaerina bakae* | *Stomiosphaerina*  *biedai* | Morphotype 1 | *Problematic* | Abundance/  Specimens number | Diversity | Ps/Po ratio |
| --- | --- | --- | --- | --- | --- | --- | --- | --- | --- | --- | --- | --- | --- | --- |
| Assemblage 3 | | 32 | 7 |  |  | 348 | 31 |  |  | 7 | 10 | 403 | 4 | 0.09 |
|  |  | 30 |  |  |  | 411 | 34 |  |  |  | 9 | 454 | 2 | 0.08 |
|  |  | 29 | 12 |  |  | 329 | 54 |  |  | 6 | 13 | 414 | 4 | 0.16 |
| Assemblage 2 | | 25 | 15 | 4 | 4 | 296 | 140 | 12 | 6 | 3 | 4 | 484 | 8 | 0.47 |
|  |  | 22 | 13 | 6 | 11 | 467 | 134 | 9 | 4 | 5 | 9 | 658 | 8 | 0.29 |
|  |  | 20 | 17 | 10 | 9 | 385 | 195 | 17 | 8 |  | 11 | 652 | 7 | 0.51 |
|  |  | 17 | 35 | 18 | 21 | 1118 | 337 | 14 | 6 | 4 | 15 | 1568 | 8 | 0.30 |
| Assemblage 1 | Sub-assemblage C | 13 |  |  |  | 123 | 46 |  |  |  | 5 | 174 | 2 | 0.37 |
|  | Sub-assemblage B | 10 |  |  |  | 1683 | 320 |  |  |  | 13 | 2016 | 2 | 0.19 |
|  |  | 8 |  |  |  | 1450 | 375 |  |  |  | 9 | 1834 | 2 | 0.26 |
|  |  | 7 |  |  |  | 1757 | 555 |  |  |  | 11 | 2323 | 2 | 0.32 |
|  |  | 6 |  |  |  | 1045 | 426 |  |  |  | 9 | 1480 | 2 | 0.41 |
|  |  | 4 |  |  |  | 1121 | 470 |  |  |  | 10 | 1601 | 2 | 0.42 |
|  |  | 3 |  |  |  | 2041 | 599 |  |  |  | 16 | 2656 | 2 | 0.29 |
|  | Sub-assemblage A | 2 |  |  |  | 27 | 8 |  |  |  | 2 | 37 | 2 | 0.30 |
